# Intrahepatic subcapsular hematoma after laparoscopic cholecystectomy in a male patient: a case report

**DOI:** 10.1093/jscr/rjae498

**Published:** 2024-08-09

**Authors:** Mamoun Aliabusunoon, Abdulrahman Baroom, Hossam Abdulghafar, Hala Alssied

**Affiliations:** Faculty of Medicine, Department of Internal Medicine, University of Gezira, Gama'a, 21111 P.O. Box 20, Wad Medani, Gezira, Sudan; Department of General Surgery, King Salman bin Abdulaziz Medical City, 8004 Sulayman Bin Othman, 42319 Mahzur, Al-Medinah Al-Munawwarah, Saudi Arabia; Department of General Surgery, King Salman bin Abdulaziz Medical City, 8004 Sulayman Bin Othman, 42319 Mahzur, Al-Medinah Al-Munawwarah, Saudi Arabia; Department of Clinical Science, Consultant of Community Medicine and Public Health, College of Medicine, Al-Rayan College, Hejrah Street P.O. Box 41411, Al-Medinah Al-Munawwarah, Saudi Arabia

**Keywords:** intrahepatic subcapsular hematoma, laparoscopic cholecystectomy, hematoma, cholecystitis

## Abstract

A 41-year-old male, with a clear medical background, underwent laparoscopic cholecystectomy for uncomplicated acute cholecystitis. No complications were observed intraoperatively. Second day after operation, the patient developed intense right upper quadrant pain, dizziness, and hypotension with a hemoglobin drop to 8.8 g/dl. Subsequently, an urgent computed tomography was done, which identified subcapsular hepatic with an intraparenchymal hematoma, and therefore, the diagnosis of intrahepatic subcapsular hematoma (ISH) was made. After fluid resuscitation and blood transfusion, the hemodynamic status became stable with no further hemoglobin decline noted. Further serial imaging was conducted and showed no signs of expansion nor intra-abdominal hemorrhage and the conservative line of management was carried on. Nine days later, the patient was discharged home. This report emphasizes the importance of close monitoring of patients who undergo LC and the possibility of ISH, although being rare, in those who report acute abdominal pain and hemodynamic instability after LC.

## Introduction

Laparoscopic cholecystectomy (LC) is considered the mainstay of treatment for symptomatic gallbladder disease as it showed a significant decrease in postoperative complications, mortality rates, and hospital stay over the past years [1]. Therefore, leading to a total transition from open surgery to laparoscopic methods. Intrahepatic subcapsular hematoma (ISH) is one of the rare and life-threatening complications after LC [1]. From 1994 till 2019, there have been only 18 cases reported [2]. Consequently, the diagnostic approach and the management of ISH after LC remain largely limited [1]. We present this case report of a patient who underwent LC and developed an ISH.

## Case report

**Figure 1:**
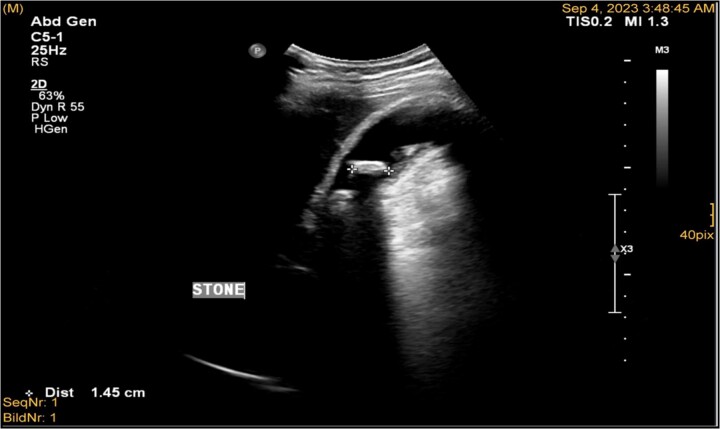
Ultrasonography demonstrated a distended gallbladder, thickened gallbladder wall measuring 6 mm, with multiple gallbladder stones, the largest one measuring approximately 1.4 cm.

**Figure 2:**
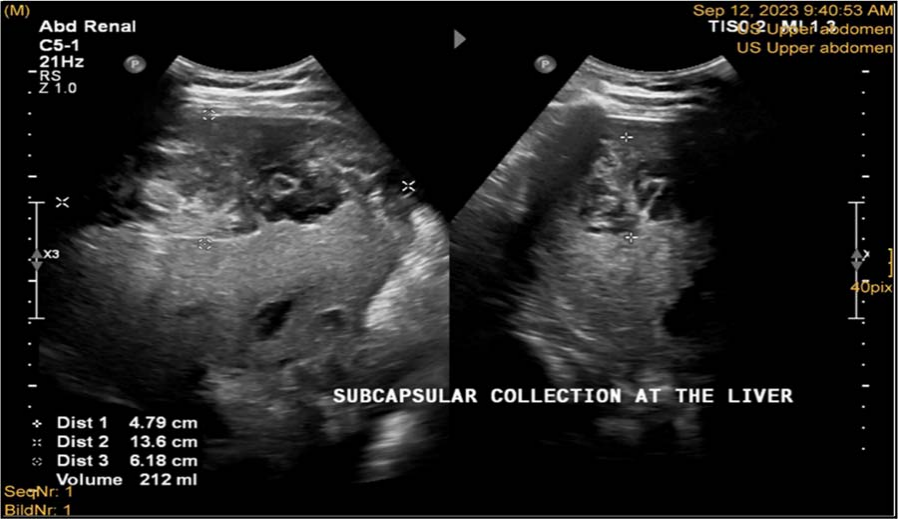
Ultrasonography postlab chole, shows right liver lobe subcapsular heterogenous density collection with liver surface being scalloped and pushed away from the capsule. Volume was about 212 ml. Morison pouch and Lino renal pouch was free.

**Figure 3:**
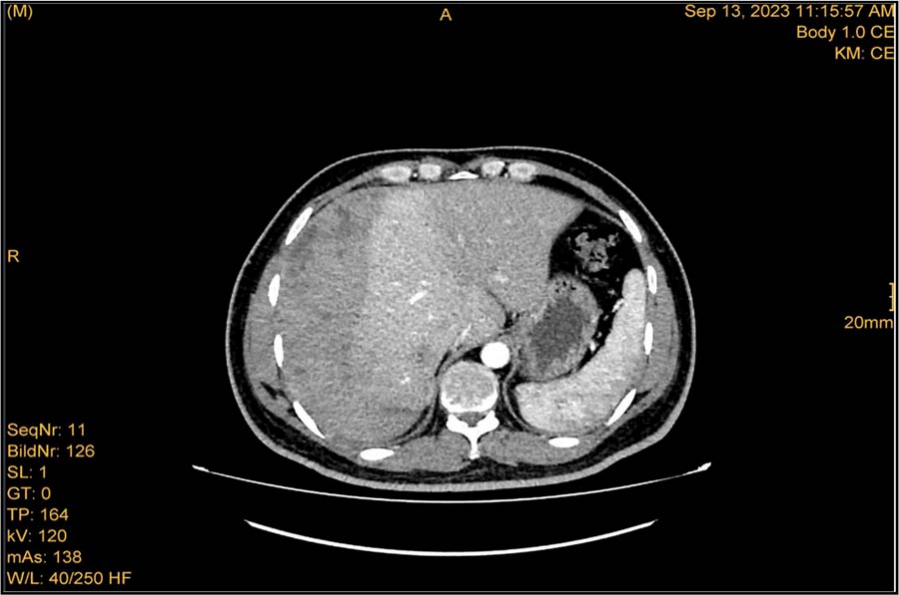
Enhanced CT scan utilizing bleeding protocol. Status post-LC identified significant ISH with internal hyperdensity measuring 7.1 × 19 × 21 cm, in its transverse, anteroposterior, and craniocaudal diameters, respectively. Adjacent hyperemic liver parenchyma with intraparenchymal hematoma noted at segment 5, measured 4.7 × 4 × 4.7 cm. Intraparenchymal hypodensities noted at segments 8 and 4b, likely represent hepatic contusions and lacerations. No evidence of active contrast extravasation.

**Figure 4:**
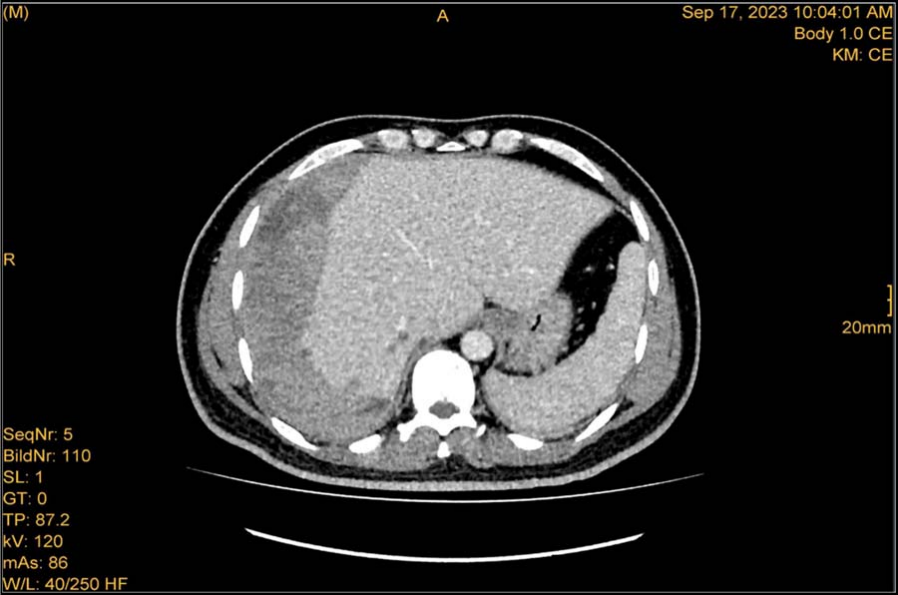
The study was done utilizing monophasic porto venous phase demonstrating intact vasculature. Relatively stable significant subcapsular and intraparenchymal hepatic hematoma with underlying parenchymal contusion and laceration. The study appears unremarkable with no new significant interval change.

A 41-year-old male with a clear medical background, presented to the Emergency Department with right upper quadrant (RUQ) abdominal pain for two days, associated with fatty dyspepsia, anorexia, nausea, and vomiting. On arrival, his heart rate was 111 bpm with a blood pressure of 128/94 mmHg. On examination, a yellowish discoloration of skin and sclera was noted with a negative murphy sign. Investigations showed elevated liver enzymes (LFT). An initial abdominal ultrasound showed a distended gallbladder with a thickened wall and multiple stones, largest measuring approximately 1.4 cm, and a common bile duct measuring 5 mm (Fig. 1). A magnetic resonance cholangiopancreatography (MRCP) identified a tiny 4-mm mid-cystic duct stone without intrahepatic biliary dilatation. After the initial management, an emergency LC was performed, which posed minimal challenges. Fortunately, no intraoperative complications were reported. A drain was kept in the subhepatic area, and the patient was kept under close observation. Second day postoperatively, the patient experienced dizziness and an intense RUQ pain despite proper analgesia. His heart rate was 89 bpm with a blood pressure of 107/64 mmHg. Investigations showed a drop in hemoglobin level from 13.8 to 9.9 g/dl. An abdominal ultrasound showed a right subcapsular heterogeneous collection with a scalloped liver surface (Fig. 2). Therefore, an initial diagnosis of ISH post-LC was considered. Following the initial fluid resuscitation and blood transfusion, a further decline in hemoglobin level was noted reaching 8.8 g/dl. Further blood transfusion was commenced achieving stabilization. Further CT scan showed subcapsular hepatic hematoma measuring 7.1 × 19 × 21 cm, in its transverse, anteroposterior, and craniocaudal diameters, respectively, with no evidence of active extravasation (Fig. 3) confirming the final diagnosis of ISH post-LC. A multidisciplinary decision was made to continue conservative management after a proper explanation of the potential risk of sudden rupture of hematoma. The patient’s clinical condition and hemoglobin level, in addition to drain output, were meticulously monitored in the general ward, ensuring early detection of deterioration. Four days later, a follow-up CT scan revealed no new significant interval changes (Fig. 4). Nine days postoperatively, the patient was discharged with a follow-up appointment, which showed almost complete resolution.

## Discussion

LC is globally accepted as the mainstay treatment of choice for symptomatic gallbladder disease with an overall morbidity of less than 7% [3]. Nevertheless, it carries serious complications with an incidence of 2.6% [4]. The most common of which include postoperative infections, bile leak, and postoperative bleeding [5]. Postoperative bleeding is notably rare with an incidence of less than 1% [6, 7]. Common sites of bleeding include gallbladder fossa, trocar placement site, cystic artery, falciform ligament, and from ruptured liver capsule [8]. Although incidence of ISH post-LC is considered rare, it poses a life-threatening hazard due to hemodynamic instability, imposing the need for special attention [9]. According to Saad *et al.*, only 18 cases of post-LC-ISH were reported in the literature between the years 1994 and 2019 [2]. All these cases were females, in contrast to ours. On account of its rarity, no indisputable cause has yet been identified but iatrogenic trauma, preoperative administration of NSAIDs and anticoagulants, anatomical variations, and hepatic hemangiomas have all been postulated as contributing factors [10–12]. The average time interval of diagnosis ranges from 6 hours to 6 weeks [1]. Our patient experienced hemodynamic instability two days postoperatively eliminating the likelihood of large ISH. About 50% of patients had hemodynamic instability on initial presentation, none were fatal [1]. Various treatment strategies were conducted according to the presentation and clinical status, ranging from conservative management, drainage under CT/US guidance, and selective embolization, to laparoscopic exploration, and laparotomy, which was indicated in seven patients [1].

In our case, however, no specific etiology was evident. His coagulation profile was normal; therefore, administration of anticoagulants postoperatively cannot be attributed to his presentation. There was no evidence of hepatic hemangiomas, additionally, neither a bleeding source nor evident injury was reported throughout the operation. Nevertheless, the possibility of a subtle capsular tear cannot be definitively ruled out. Unsurprisingly, the exact etiology remained unaccounted for in seven documented cases [1]. In absence of the mentioned factors, we postulate that the etiology was due to intraoperative manipulation, which led to spontaneous hemorrhage.

The management of ISH depends on the patient’s status and the size of hematoma. Our patient’s positive response to conservative management favored its continuity, which can be considered if the hematoma is confined to the liver capsule and small in size [13]. Hematoma expansion and rupture should be kept in mind if a conservative approach is commenced. The substantial need for intervention either laparoscopically or conventionally must be considered based on clinical and radiological parameters [2, 13]. In case of hemodynamic instability, guided drainage of the hematoma is a feasible choice if the size is significant and when signs of co-infection exist [14]. A previously reported case also described the use of a selective embolization of an active bleeder in the right hepatic artery, followed by surgical evacuation if failed [10]. Timely and appropriate imaging plays a crucial role in diagnosing and guiding the management of ISH [9].

## Conclusion

ISH is a rare yet potentially life-threatening post-LC complication. This report emphasizes the importance of close monitoring of patients who undergo LC and the possibility of ISH, although being rare, in those who report acute abdominal pain and hemodynamic instability after LC.
